# CXCL1 promotes cell migration in hepatocellular carcinoma by regulating the miR-30b-5p/ICAM-1 axis

**DOI:** 10.7150/jca.95816

**Published:** 2024-07-22

**Authors:** Yi-Hsin Chen, Chih-Chun Chu, Augusta I-Chin Wei, Ju-Fang Liu, Hong-Shiee Lai

**Affiliations:** 1 Division of Pediatric Surgery, Department of Surgery, Tri-Service General Hospital, National Defense Medical Center, Taipei, Taiwan.; 2 Department of Emergency Medicine, Tri-Service General Hospital, National Defense Medical Center, Taipei, Taiwan.; 3 Graduate Institute of Clinical Medicine, College of Medicine, National Taiwan University, Taipei, Taiwan.; 4 School of Oral Hygiene, College of Oral Medicine, Taipei Medical University, Taipei, Taiwan.; 5 Translational Medicine Center, Shin-Kong Wu Ho-Su Memorial Hospital, Taipei, Taiwan.; 6 Department of Medical Research, China Medical University Hospital, China Medical University, Taichung, Taiwan.; 7 Department of Surgery, College of Medicine, National Taiwan University Hospital and National Taiwan University, Taipei 10002, Taiwan.; 8 Department of Surgery, Buddhist Tzu Chi Medical Foundation, Hualien Tzu Chi Hospital, Hualien 97002, Taiwan.

**Keywords:** Human hepatocellular carcinoma (HCC), metastasis, CXCL1, ICAM-1, miR-30b-5p

## Abstract

Hepatocellular carcinoma (HCC) is a highly lethal cancer with a growing global incidence and is often associated with poor prognosis due to its tendency to metastasize. Intercellular adhesion molecule (ICAM) 1 is a transmembrane protein found in various cancer cells and is associated with the spread of cancer and poor prognosis. Chemokine (C-X-C motif) ligand 1 (CXCL1) is a chemokine that significantly affects the cell motility of various cancers. However, the role of CXCL1 in ICAM-1 expression and in metastasis of hepatocellular carcinoma remains unclear. We determined that CXCL1 expression is positively and significantly associated with advanced-stage tumors in the HCC tissue array. Kaplan-Meier analysis revealed worse overall survival rates in the high CXCL1 expression group, suggesting its potential as a biomarker for cancer progression and stimulating hepatocellular carcinoma cells with CXCL1 enhanced migration abilities by upregulating ICAM-1 expression. CXCL1 was shown to enhance ICAM-1-dependent cell motility by inhibiting miR-30b-5p. This study provides novel evidence that CXCL1 could serve as a therapeutic target for metastasis in hepatocellular carcinoma.

## Introduction

Human hepatocellular carcinoma (HCC) is the most prevalent form of liver cancer and ranks as the third leading cause of cancer-related deaths worldwide 1, 2. HCC is a highly malignant tumor that can lead to both intrahepatic and extrahepatic metastases 3. Extrahepatic metastasis, which occurs in one-third of HCC patients, is associated with a poor prognosis. The average survival duration of approximately 8.1 months when liver cancer metastasizes beyond the liver 4. The lungs, regional lymph nodes, and bones are the most common sites of extrahepatic metastasis 5. The etiological risk factors for HCC include infections with hepatitis B and C viruses, fatty liver disease, alcohol consumption, and exposure to environmental toxins 6. Treatment strategies for HCC depend on the stage of tumor. Surgical resection, liver transplantation, and ablation therapies remain effective for early-stage HCC, while embolization and radiation therapy are used in the intermediate stages. Advanced-stage HCC is treated using a variety of approaches, including chemotherapy, targeted therapy, immunotherapy, and gene therapy 7. However, the prognosis for patients with HCC is often grim due to the high rate of metastasis 3. Therefore, the challenge in treating HCC arises from metastasis.

Intercellular adhesion molecule 1 (ICAM-1; CD54), a cell surface glycoprotein, is known to be involved in cancer metastasis 8. ICAM-1 facilitates cell-cell interactions by promoting adhesion and assists in the movement or retention of cells within the extracellular matrix. ICAM-1 correlates to larger tumor size, metastasis, and recurrence in HCC, leading to a poorer prognosis 9. Moreover, ICAM-1 is more abundant in hepatocytes within cancerous regions compared to noncancerous areas 10. An increase in ICAM-1 expression may be directly associated with a poor prognosis in HCC patients 11. In tumor progression, abnormal microRNAs (miRNAs) are related to cancer cell mobility and migration and regulate ICAM-1 expression 12. Therefore, investigating the activation mechanism of ICAM-1 and the involvement of miRNAs will help us understand the processes of cellular migration in HCC metastasis.

Evidence suggests that chemokines are crucial in neoplastic transformation, cancer progression, and angiogenesis 13. Chemokine (C-X-C motif) ligand 1 (CXCL1/GROα), a proangiogenic CXC-type chemokine, is found in various cancer types, including melanoma, breast, lung, pancreatic, colorectal, and prostate cancers 14, 15. Furthermore, several studies have shown a correlation between the overexpression of CXCL1 and poor prognosis in cancer 15-17. CXCL1 has been identified as a candidate gene that could function as a clinically relevant biomarker in HCC 18. However, the effect of CXCL1 on cancer metastasis in HCC has been poorly delineated.

This study focused on the role of microRNAs in CXCL1-mediated ICAM-1 expression and cell motility in human hepatocellular carcinoma. We found that CXCL1 levels were significantly higher in HCC tissues compared to healthy liver tissues. Our findings indicate that CXCL1 enhances ICAM-1-driven cell migration in HCC. The downregulation of miR-30b-5p synthesis was implicated in the CXCL1-induced upregulation of ICAM-1 production and migration. These findings suggest that CXCL1 could be a promising target for metastatic human hepatocellular carcinoma.

## Materials and Methods

### Materials

The human recombinant CXCL1 protein was purchased from PeproTech (Rocky Hill, NJ, USA). This study used polyclonal antibodies specific for CXCL1, ICAM-1, and β-Actin purchased from Santa Cruz Biotechnology (Santa Cruz, CA, USA). Lipofectamine 3000 was purchased from Invitrogen (Carlsbad, CA, USA). All other chemicals were purchased from Sigma-Aldrich (St. Louis, MO, USA).

### Immunohistochemistry (IHC) staining

A human HCC tissue array was obtained from US Biomax (Derwood, Maryland, USA). The sections were deparaffinized using xylene and rehydrated with ethanol for immunohistochemistry (IHC) staining. The sections were immunoassayed with CXCL1 antibody (1:300) overnight, followed by an incubation with secondary antibody (1:200) for 1 h at room temperature. Finally, all tissues were stained with 3,3-diaminobenzidine and photographed using a Nikon ECLIPSE Ti microscope (NIS Elements AR 5.02.01). The scoring of IHC images incorporated the proportion of positive detection, quantified as a percentage ranging from 0 to 100 using Image J 1.52a software, and the staining intensity, graded on a scale from 0 to 3+. This dual-parameter assessment yielded a composite score with a potential range of 0 to 300.

### Analysis of Messenger RNA (mRNA) expression profiles from the GEO database

Screening of the Gene Expression Omnibus (GEO) datasets revealed microarrays related to HCC: GSE40367 and GSE46408. The GSE46408 profile is a genome-wide comparison of gene expression, which includes differentially expressed genes in 6 healthy liver tissues and 6 HCC samples. The GSE40367 profile is a genome-wide comparison of gene expression, which includes differentially expressed genes in 5 healthy liver tissues and 10 HCC samples.

### Kaplan-Meier plotter

The KM plotter (https://kmplot.com/analysis/index.php?p=background) analyzed datasets from over 30,000 samples from 21 different tumor types. Furthermore, the statistical analysis involved the use of Cox proportional hazards regression and the computation of the False Discovery Rate. We identified CXCL1 in liver hepatocellular carcinoma (LIHC). We conducted two survival analyses to evaluate the effect of non-coding RNA expression levels on patients' overall survival (OS) and disease-specific survival (DSS). Additionally, we obtained *p*-values from log-rank tests to determine the significance of survival rates.

### Analysis of UALCAN database

UALCAN (https://ualcan.path.uab.edu/analysis.html) is a resourceful website for explorers to access Level 3 RNA-seq data from The Cancer Genome Atlas (TCGA) and perform gene expression and survival analysis in 31 different tumor types. The data was statistically analyzed using Welch's t-test to determine the significance of differences in expression levels between normal and primary tumors and tumor subgroups based on individual cancer stages and tumor grade. Each box-whisker plot displays the interquartile range (IQR), which includes the minimum, 1st quartile, median, 3rd quartile, and maximum values. In the statistical analysis, we used the descriptive PERL module to calculate IQR values after filtering outliers.

### Cell culture

Human HCC cell lines, including HepG2, Hep3B, Huh7, and HepG2/C3A, were acquired from the American Type Cell Culture Collection (Manassas, VA, USA). Cells were cultured and maintained in Dulbecco's Modified Eagle Medium (DMEM) supplemented with 10% fetal bovine serum (FBS) and penicillin (100 U/mL)/streptomycin (100 µg/mL) at 37 °C with 5% CO_2_.

### Cell migration assay

Following the established protocol, cell migration was assessed using a 24-well cell transwell with 8.0 µm-pore polycarbonate membrane inserts (Corning, NY, USA). Cells were stimulated with CXCL1 in the lower chamber. After 24 h, the migrated cells were fixed with 3.7% formaldehyde and stained with crystal violet. Cell migration was quantified using a microscope by counting the number of stained cells.

### Western blot analysis

The HepG2, Hep3B, Huh7, and HepG2/C3A cells were treated with CXCL1 (0, 10, and 30 ng/mL) for 24 h and then with RIPA lysis buffer containing a 1% cocktail of protease inhibitors at 4°C. The protein samples (40 μg/well) were separated using 10-15% SDS-PAGE and transferred onto a polyvinylidene difluoride (PVDF) membrane (Millipore, USA). Membranes were incubated with primary antibodies against CXCL1, ICAM-1, and β-Actin at 4 °C overnight, followed by HRP-conjugated secondary antibodies (1:25,000) at 25 °C for 2 h. Protein expression levels were visualized using SuperSignal West Pico Chemiluminescent Substrate (Thermo Fisher Scientific, Waltham, MA, USA).

### The quantitative real-time reverse transcription-polymerase chain reaction (qPCR)

Total RNA was extracted from HCC cells using a TRIzol kit (MDBio Inc., Taipei, Taiwan). Total RNA (2 µg) was reverse transcribed into cDNA using oligo (dT) primer 19, 20. The quantitative real-time PCR (qPCR) analysis used Taqman^®^ one-step PCR Master Mix (Applied Biosystems, CA, USA). cDNA (2 µL) was added per 25 µL reaction with sequence-specific primers and Taqman^®^ probes. Sequences for all target gene primers and probes, with GAPDH as the internal control (Applied Biosystems, CA, USA). qPCR assays were performed in triplicate using a Step One Plus sequence detection system. The cycling conditions were 10-min polymerase activation at 95°C, followed by 40 cycles at 95°C for 15 s and 60°C for 60 s. The threshold was set above the non-template control background and within the linear phase of target gene amplification to calculate the cycle number at which the transcript was detected (denoted as CT).

### Establishment of migration-prone sublines

A cell culture insert system was used to select subpopulations of HepG2 cells based on their differential migration ability. Cell migration was allowed to occur for 24 h. Cells that penetrated the pores and migrated to the underside of the filters were then trypsinized and harvested for a second selection round. All wild-type cells that did not migrate through the membrane pores were designated as M0. Following 5 rounds of selection, the migrated sublines were designated as M5.

### Analysis of the LinkedOmics database

Our study involved a detailed examination of the LinkedOmics database (https://www.linkedomics.org/login.php), a comprehensive resource offering multi-omics data across all 32 types of cancer included in The Cancer Genome Atlas (TCGA), along with various clinically relevant datasets. Using the Linkfinder tool, we conducted a statistical analysis of CXCL1 co-expression employing the HiSeq RNA platform and Pearson's correlation coefficient. This process yielded volcano plots and heat maps that distinctly showcase significant differential expression and gene correlations. Furthermore, our study explored the relationship between CXCL1 expression, clinicopathological characteristics, overall survival (utilizing Cox regression analysis), and the histological types of cancer, as revealed by mass spectrometry (MS)-based global proteomics data. We also utilized the Linkinterpreter module to identify a functional association module, focusing on the analysis of Gene Ontology biological processes (GO_BP), Gene Ontology pathways, and Kyoto Encyclopedia of Genes and Genomes (KEGG) pathways. This analysis adhered to a strict ranking criterion of a False Discovery Rate (FDR) less than 0.05, with 1000 simulation runs being conducted.

### CBioPortal database

The c-Bio Cancer Genomics Portal (https://www.cbioportal.org/) provides access to multidimensional cancer genomics datasets, including over 5000 tumor samples across 20 different types of cancer for exploration. We identified a single CXCL1 gene in hepatocellular carcinoma (TCGA, Firehose Legacy) using 379 samples. The data shows a co-occurrence of CXCL1 gene in HCC. Clinical data from datasets provide an overview of genetic alterations per sample (366 samples/patient) in CXCL1.

### Cell transfection

The ICAM-1 siRNA (5'-AAACAACCGGAAGGUGUAUGA-3') and control siRNAs were commercially purchased from Santa Cruz Biotechnology (Santa Cruz, CA, USA). The sequence of CXCL1 shRNA (5'-GCACATCTGTTTTGTAACT-3') and the control shRNA sequence (5'-TTCTCCGAACGTGTCACGT-3') were commercially purchased from the National RNAi Core Facility of Academia Sinica (Taipei, Taiwan). miR-30b mimic and control mimic were purchased commercially from AllBio Science (Taipei, Taiwan). The specific sequence for the has-miR-30b-5p mimic is 5'-UGUAAACACUCUCAGCU-3', and the control mimic is 5'-UUGUACUACACAAAAGUACUG-3'. Cells were transfected with siRNAs (at a final concentration of 100 nM) using Lipofectamine 3000 (Invitrogen; Carlsbad, CA, USA) according to the manufacturer's instructions.

### Statistical analysis

All data were presented as the mean ± standard deviation (SD) from 3 experiments. Statistical differences between groups were calculated using Student's t-test and one-way analysis of variance, followed by Dunnett's post hoc tests. Statistical significance was considered at *p* < 0.05.

## Results

### Elevated levels of CXCL1 expression in patients with metastatic hepatocellular carcinoma

**Figure 1:**
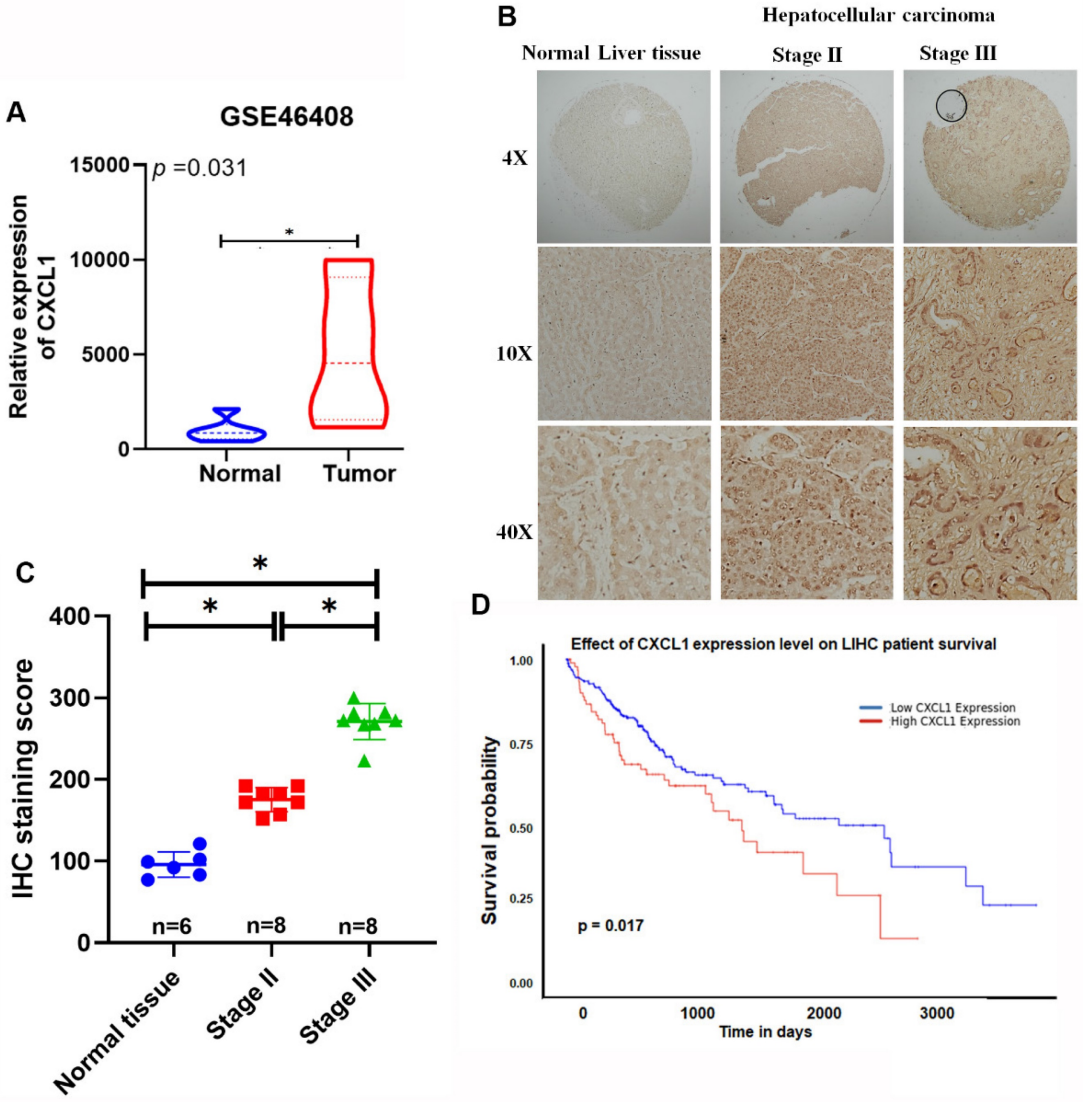
** Overexpression of CXCL1 is correlated with poor prognosis in hepatocellular carcinoma.** (A) Levels of CXCL1 gene expression in the normal liver tissue and hepatocellular carcinoma using GEO microarray 46408. (B) Representative images of immunohistochemical staining of CXCL1 expression in normal human liver tissue and hepatocellular carcinoma. (C) IHC scores for CXCL1 expression in different stages of hepatocellular carcinoma and normal tissues. (D) Kaplan-Meier survival analysis of the associations between high or low plasma levels of CXCL1 expression and overall survival of HCC patients. Results are expressed as mean ± SD of three independent experiments. * *p* < 0.05 compared to control.

CXCL1 promotes the progression of various cancers, including breast, lung, pancreatic, colorectal, and prostate cancers 14, 15. However, its role in HCC remains unknown. We analyzed gene expression profiling data from the Gene Expression Omnibus (GEO) database (GSE46408) for CXCL1. Significantly higher levels of CXCL1 expression were found in HCC tissues compared to healthy liver tissue samples (Figure 1A). Furthermore, tissue array data showed elevated levels of CXCL1 expression in advanced HCC samples compared to healthy liver samples (Figure 1B-C). The analysis of these results revealed significantly higher levels of CXCL1 expression in the advanced-stage (stage III) tumors compared to the stage II tumors and normal tissue samples (Figure 1C). This suggests a positive correlation between the levels of CXCL1 expression and the progression of HCC cancer. Furthermore, the Kaplan-Meier overall survival rates were significantly poorer for the group with high CXCL1 expression compared to the group with low CXCL1 expression (Figure 1D). Our findings emphasize the association between elevated CXCL1 levels and adverse outcomes in HCC.

### High expression of CXCL1 is associated with the ability of migration in hepatocellular carcinoma

**Figure 2:**
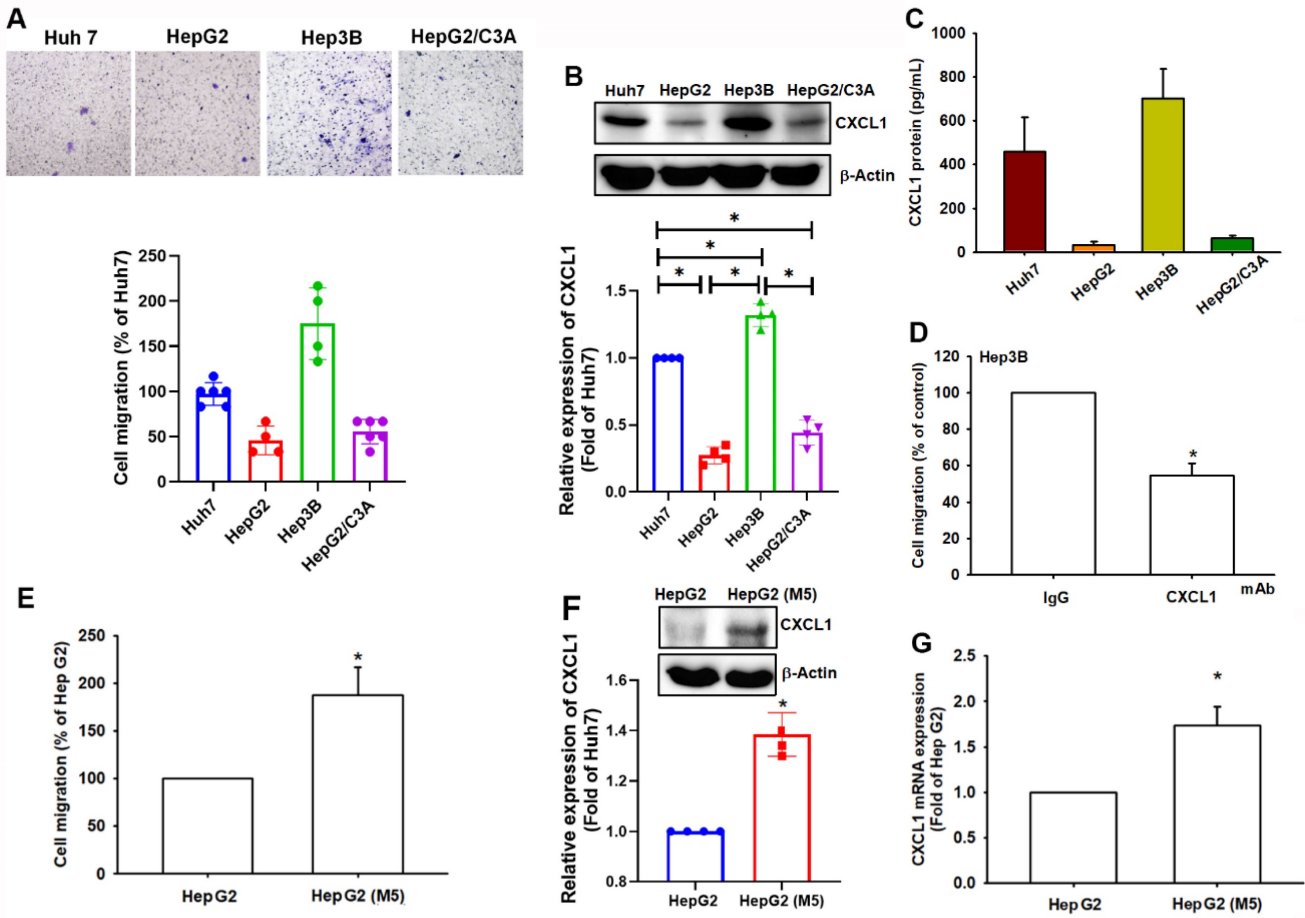
** CXCL1 upregulation is correlated with cell migration in hepatocellular carcinoma.** (A) The cell migration ability of the HCC cell lines Huh7, HepG2, Hep3B, and HepG2/C3A was assessed using the Transwell assay. (B-C) Total protein was collected from the indicated cell lines, and CXCL1 expression was detected using Western blotting and ELISA. (D) Quantified result of cell migration with CXCL1 neutralizing antibody. (E-G) Migratory ability and CXCL1 expression of the indicated cells (HepG2 and HepG2-M5) were examined by Transwell, immunoblotting assay, and qPCR. Results were expressed as mean ± SD of three independent experiments. *p < 0.05 compared with the control group of each experiment.

We compared the migratory ability of human HCC cell lines, including Huh7, HepG2, Hep3B, and HepG2/C3A, which showed varying migratory abilities. Hep3B and Huh7 cells demonstrated more significant migratory abilities than HepG2 and HepG2/C3A cells (Figure 2A). Western blot analysis and ELISA assay revealed higher levels of CXCL1 expression in Hep3B and Huh7 cells compared to HepG2 and HepG2/C3A cells (Figure 2B-C). The cell migration activity was inhibited when the cells were treated with CXCL1 neutralizing antibody in Hep3B cells (Figure 2D). To investigate the correlation between CXCL1 levels and the migratory capacity of HCC cells, we selectively cultured HepG2 cells with higher mobility to develop a subcloned cell line that exhibits enhanced migration tendencies, termed HepG2-M5 (Figure 2E). This process involved the specific selection of cells exhibiting greater migratory characteristics. In Western blot and qPCR analysis, the expression levels of CXCL1 were higher in HepG2-M5 cells compared to HepG2 cells (Figure 2F-G). This data demonstrates that the levels of CXCL1 expression in human HCC support the enhanced migratory properties.

### CXCL1 promotes cell migration in hepatocellular carcinoma cells

**Figure 3:**
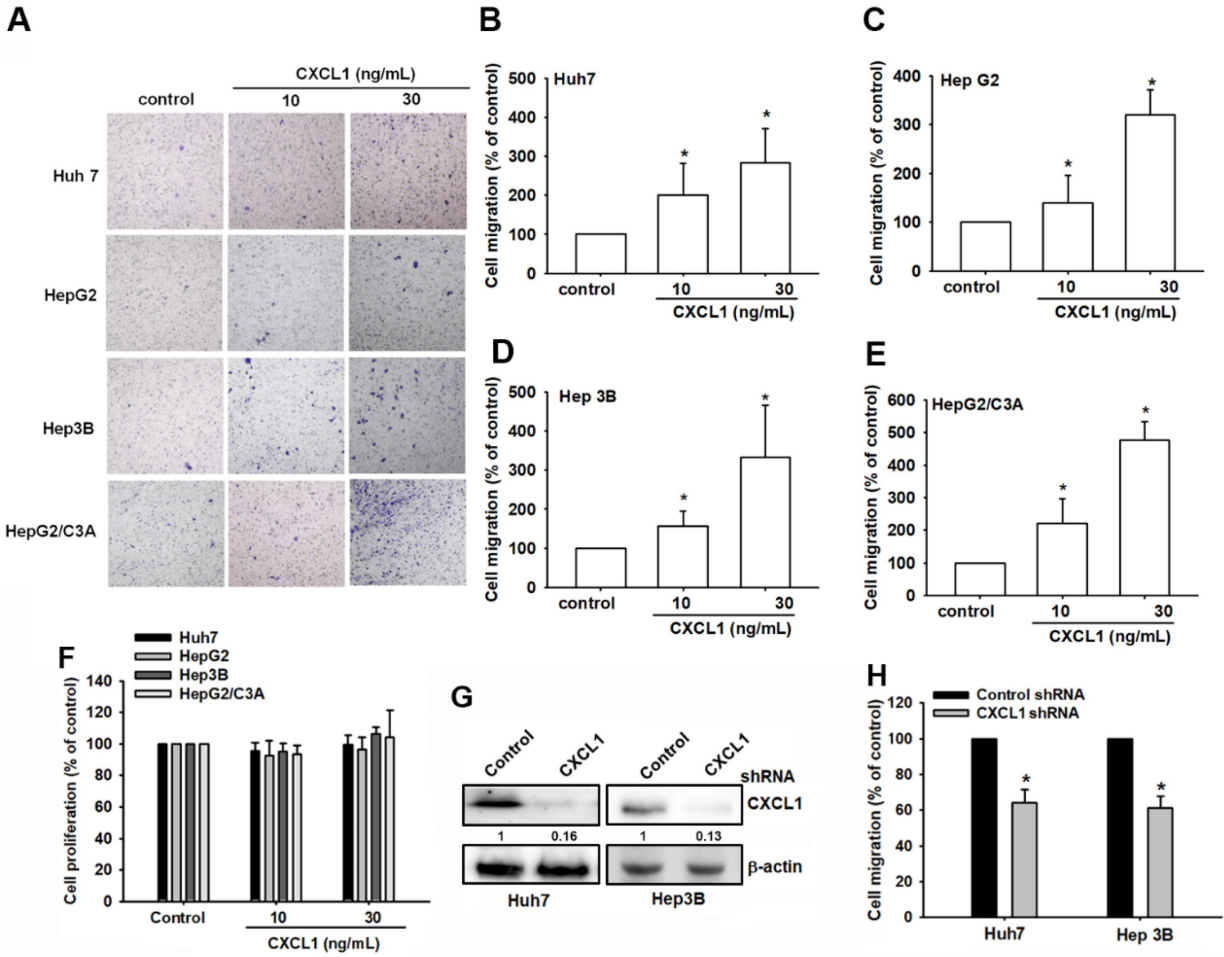
** CXCL1 upregulates human HCC cell migration.** (A-E) HCC cells were incubated with different concentrations of CXCL1 for 24 h; then, cell migration was assessed using the *in vitro* Transwell assay. (F) HCC cells were incubated with different concentrations of CXCL1 for 24 h; then, cell proliferation was assessed using the CCK8 assay. (G-H) Cells were treated with a CXCL1 shRNA; protein expression and cell migration were examined. Results are expressed as the mean ± SD of three independent experiments. *p < 0.05 as compared with controls.

We then directly used the Transwell assay, a well-established model for examining cell migration following CXCL1 treatment in hepatocellular carcinoma cells. Huh7, HepG2, Hep3B, and HepG2/C3A cell lines were exposed to various concentrations of CXCL1. Cell migration activities increased in a dose-dependent manner (Figure 3A-E). Meanwhile, stimulation with CXCL1 did not affect the proliferative capacity of oral cancer cells (Figure 3F). Knockdown of CXCL1 in Huh7 and Hep3B cells, which express CXCL1 at the highest levels, resulted in reduced migration of hepatocellular carcinoma cells (Figure 3G-H). This suggests that CXCL1 promotes the migration of hepatocellular carcinoma cells.

### CXCL1 exhibits a positive gene correlation with ICAM in various functional modules

**Figure 4:**
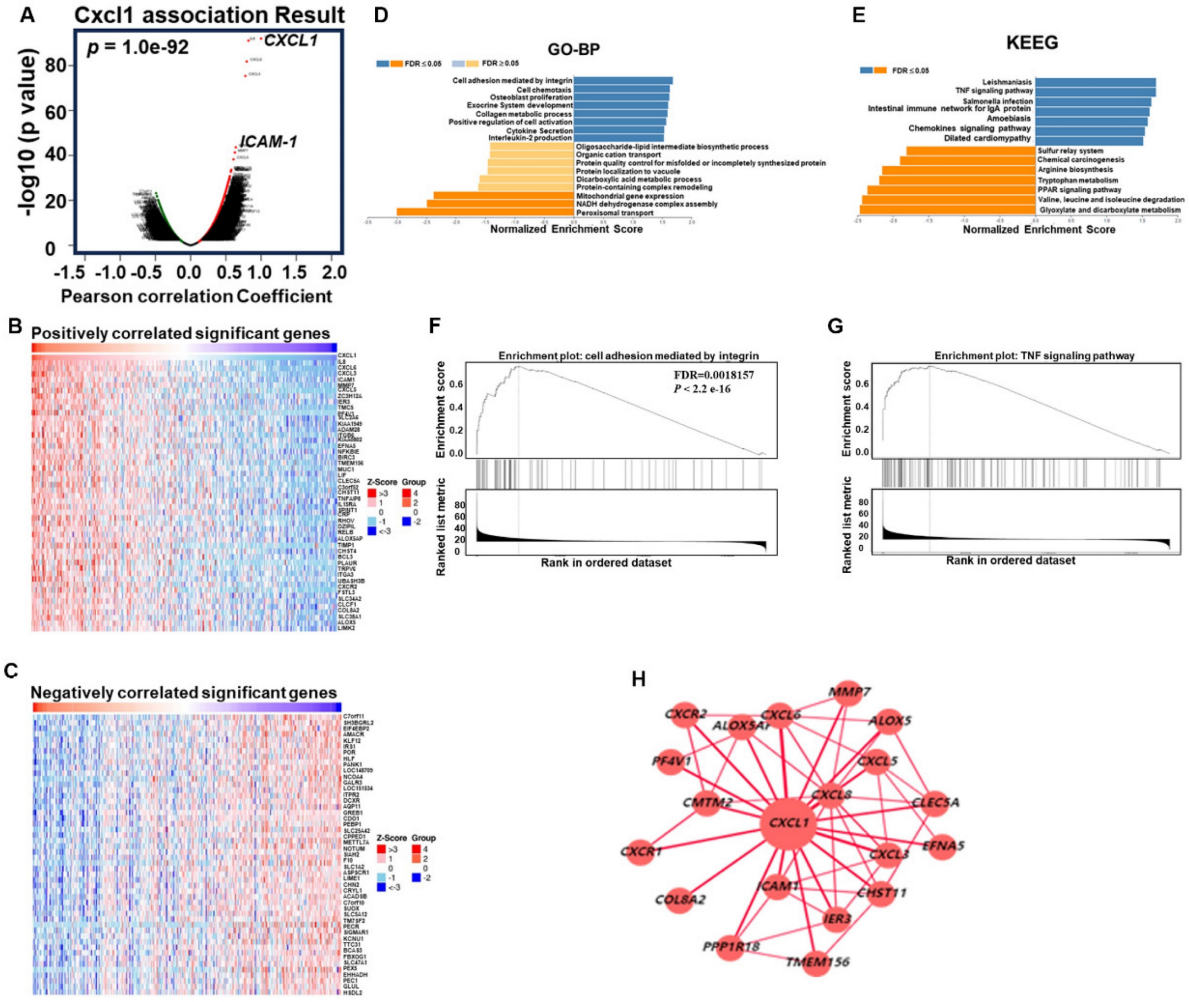
** Function of CXCL1 in HCC from LinkedOmics.** (A) CXCL1 association results. (volcano graph). (B) Positively correlated significant genes (heat map). (C) Negatively correlated important genes (heat map). (D) GO terms for biological process and (E) KEGG pathways. (F-G) GO and KEGG pathways showed that CXCL1 is involved in multiple signals, such as the integrin and TNF signaling pathways. (H) CXCL1-based regulatory schematic.

**Figure 5:**
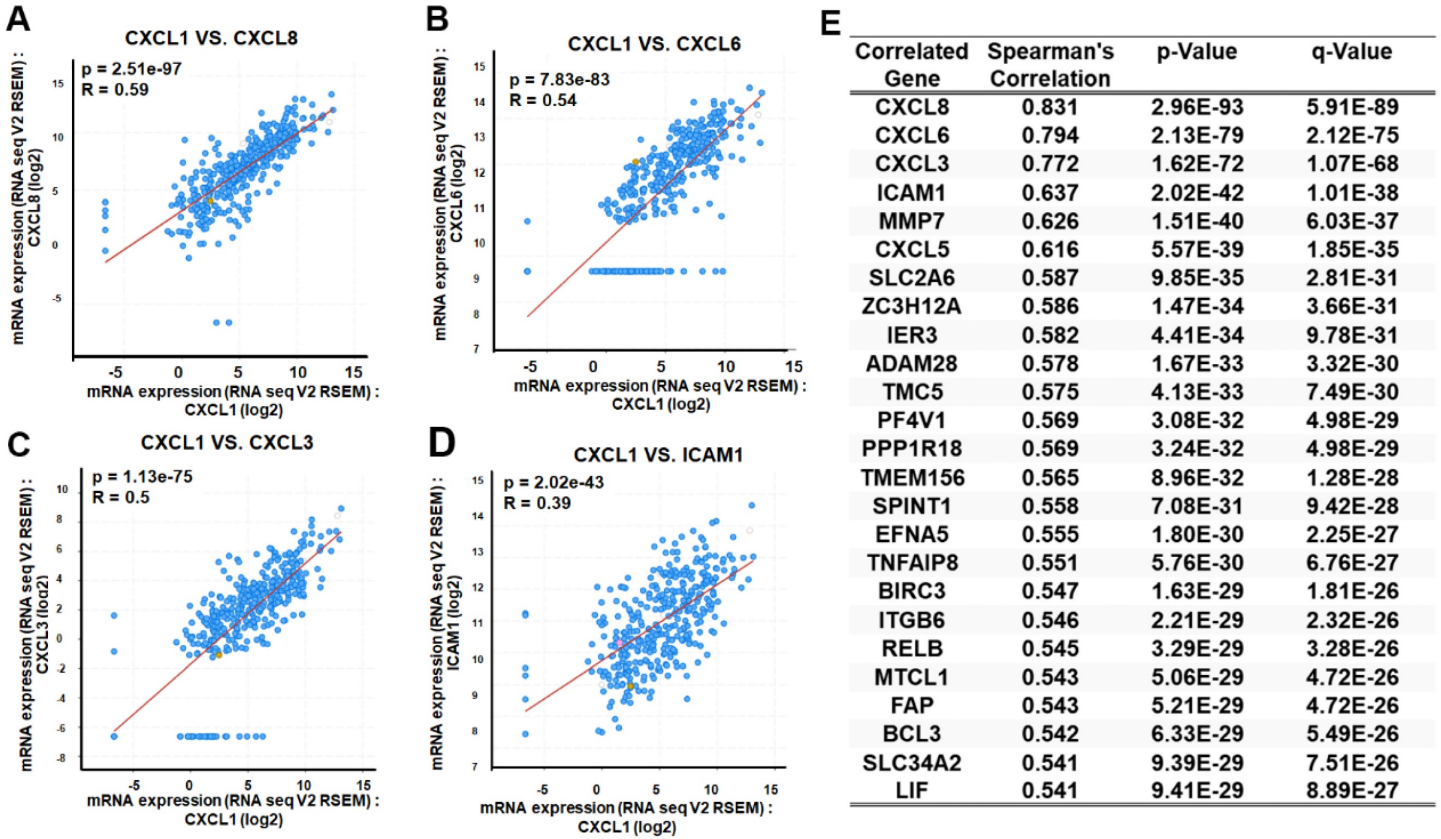
** Exploration of CXCL1 expression correlated genes using cBioPortal.** The scatter plot illustrated highly correlated genes from the TCGA-LIHC database through Spearman's correlation analysis of CXCL1 expression with (A) CXCL8, (B) CXCL6, (C) CXCL3 and (D) ICAM-1. (E) Table is the top 25 genes ranked by CXCL1 gene correlation.

As mentioned earlier, we evaluated the biological process involving CXCL1 in HCC. We utilized the functional modules and pathway enrichment analysis workflow in the LinkedOmics platform to identify several modules associated with CXCL1. We used the LinkFinder module to analyze the co-expression of CXCL1 and examined the co-expression pattern in the LIHC cohort using Pearson's correlation coefficient. In Figure 4A, the volcano plot illustrates that out of 12,919 genes (red dots), there was a significant positive correlation with CXCL1. In comparison, 7,004 genes (green dots) showed a significant negative correlation (FDR < 0.01, t-test followed by multiple testing correction). The heatmap shows the top 50 genes that exhibited substantial positive and negative correlations with CXCL1 (Figure 4B-C). Furthermore, we observed biological networking within a significant Gene Ontology (GO) module using gene set enrichment analysis (GSEA). This module exhibited co-expressed genes involved in cell adhesion mediated by integrin, with CXCL1 being a top-ranking functional process (Figure 4D and 4F). Next, the Kyoto Encyclopedia of Genes and Genomes (KEGG) pathway analysis revealed enrichment in the TNF signaling pathway (Figure 4E and 4G) involving various genes. The top-ranking 10 genes in the GO and KEGG pathways are shown in Figure 4H. In addition, CXCL1 exhibited a strong correlation with CXCL8 (*p < 0.001,* rs=0.831), CXCL6 (*p < 0.0001,* rs=0.794), CXCL3 (*p < 0.001,* rs=0.772), and ICAM (*p < 0.001,* rs=0.637) as determined by Spearman's correlation analysis in the cBioPortal online platform (Figure 5A-E). Consequently, we used LinkedOmics datasets and the cBioPortal platform in HCC, which revealed robust correlations between CXCL1 and genes from the C-X-C family or adhesion genes (ICAM-1).

### CXCL1 upregulates cell migration through intercellular adhesion molecule 1 (ICAM-1)

**Figure 6:**
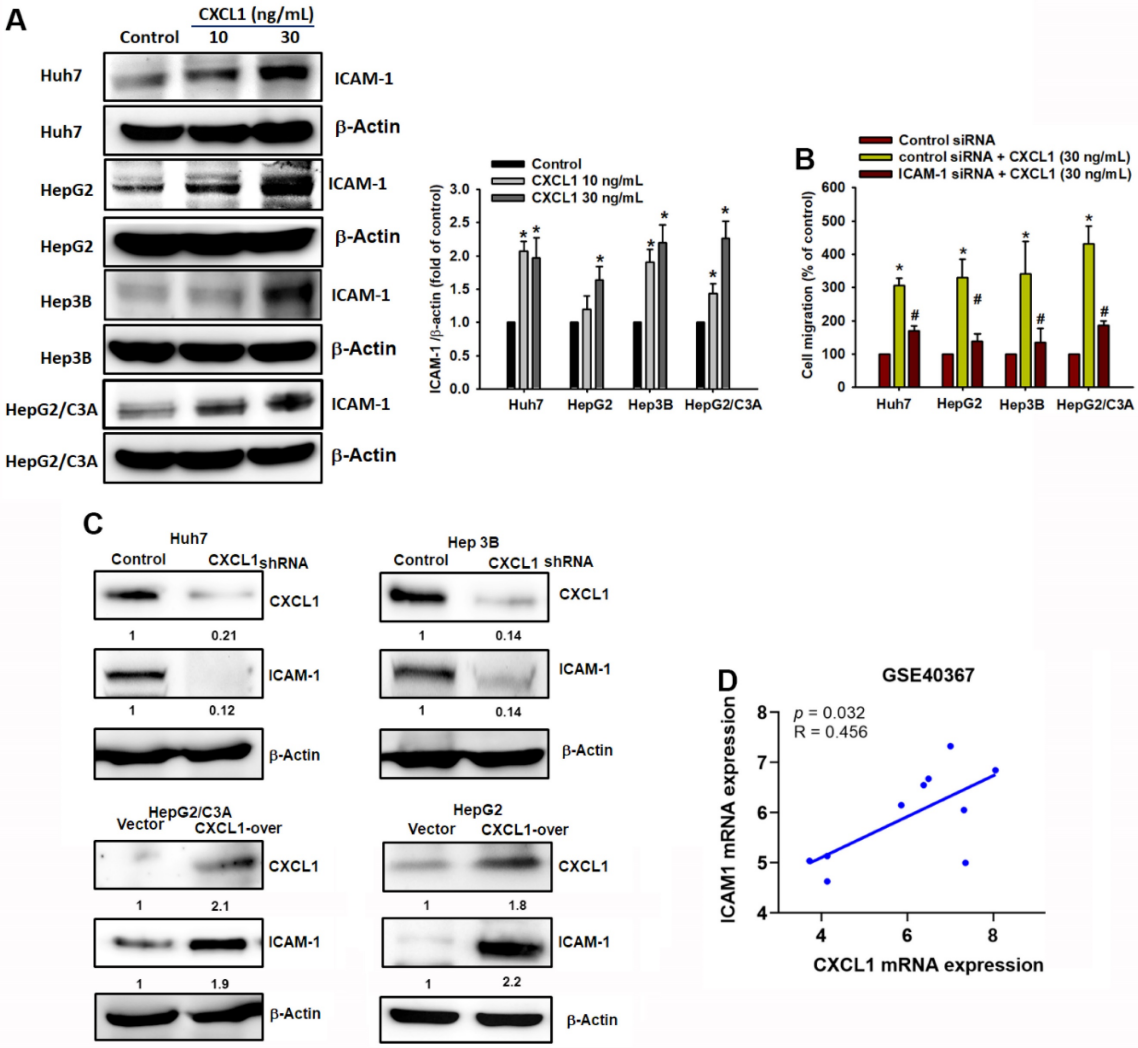
** CXCL1 activates tumor cell migration via the ICAM-1 expression in human HCC cells.** (A) Protein expression of ICAM-1 with concentration-depended CXCL1 treatment. (B) Cells were treated with ICAM-1 siRNA and then stimulated with CXCL1 (30 ng/mL); the cell migration was examined. (C) Cells were transfected with CXCL1 plasmid or shRNA, then CXCL1 expression was measured by Western blot. (D) Correlation analysis of CXCL1 and ICAM-1 expression using the GSE40367 database. Results are expressed as the mean ± SD of three independent experiments. *p < 0.05 as compared with controls; # *p* < 0.05 compared with the CXCL1-treated group.

Cell adhesion molecules play a significant role in cell migration, cell-matrix interactions, and tumor dissemination 21, 22. The expression of adhesion molecules facilitates the interaction between tumor cells and the surrounding stroma, thereby promoting tumor metastasis 23. This study investigated the role of ICAM-1 in HCC cells. Treatment with CXCL1 significantly increased the expression of ICAM-1 protein (Fig. 6A). To confirm the role of ICAM-1 in HCC cell migration driven by CXCL1, we transfected HCC cells with ICAM-1 siRNA and determined that ICAM-1 siRNA resulted in the suppression of CXCL1-induced cell migration (Figure 6B). In addition, overexpression of CXCL1 in HepG2 and HepG2/C3A cells (which have the lowest CXCL1 expression) or the knockdown of CXCL1 in Huh7 and Hep3B cells (which have the highest CXCL1 expression), respectively, promoted and reduced ICAM-1 protein expression in hepatocellular carcinoma cells (Figure 6C). Furthermore, we conducted GEO database (GSE40367) to investigate the correlation between CXCL1 and ICAM-1 in HCC patients. Figure 6D showed a strong positive correlation between the gene expression of ICAM-1 and CXCL1 in HCC (*p* = 0.032, *r* = 0.456). These findings indicate that the increased HCC cell migration mediated by CXCL1 is positively associated with high ICAM-1 expression.

### miR-30b-5p suppresses CXCL1-induced ICAM-1 expression and motility in hepatocellular carcinoma

**Figure 7:**
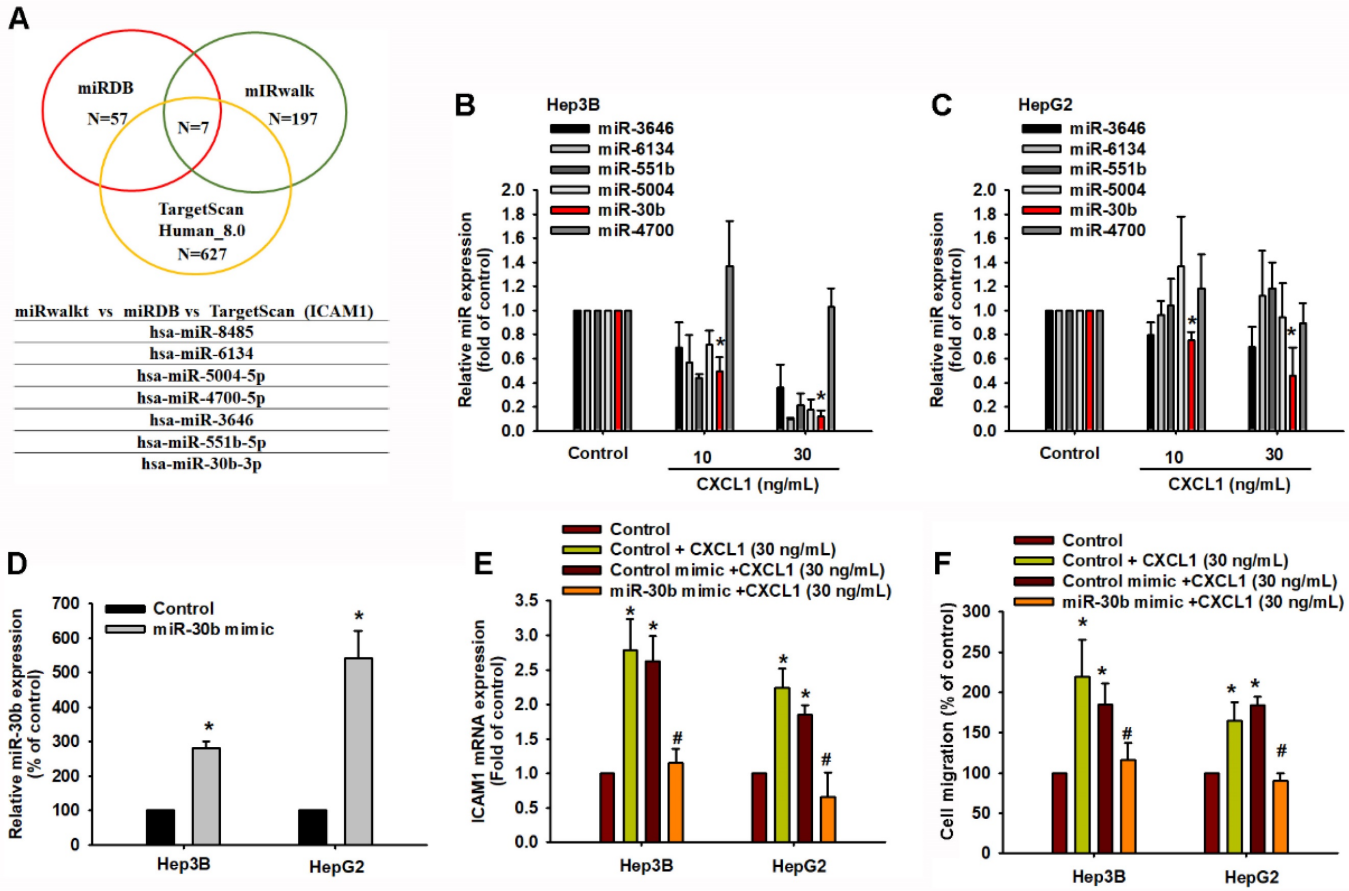
** miR-30b-5p downregulates CXCL1-mediated ICAM-1 expression and cell migration.** (A) The miRDB, miRwalk, and TargetScan computational software were used to identify potential miRNAs that bind to ICAM-1. (B-C) Cells were treated with CXCL1 for 24 h and miRNAs expression was examined by qPCR. (D) Cells were transfected with control miRNA mimic and miR-30b-5p mimic (20 nM), and the expression of miR-30b-5p was analyzed by qPCR. (E-F) Cells were transfected with control miRNA mimic and miR-30b-5p mimic and then stimulated with CXCL1 (30 ng/mL); the ICAM-1 expression and cell migration were examined. Results are expressed as the mean ± SD of three independent experiments. *p < 0.05 as compared with controls; # *p* < 0.05 compared with the CXCL1-treated group.

To further analyze potential miRNAs targeting ICAM-1, we initially used open-source software (miRWalk, TargetScan, and miRDB) to identify the miRNAs that regulate ICAM-1 expression. Among these databases, miRDB contained 57 miRNAs, miRWalk had 197 miRNAs, and TargetScan contained 627 miRNAs predicted to bind to ICAM-1 mRNA (Figure 7A). We intersected these sets and found that 7 miRNAs were involved (Figure 7A). The qPCR results indicated that hsa-miR-30b-5p was significantly downregulated compared to the others after concentration-dependent CXCL1 treatment in Hep3B and HepG2 cells (Figure 7B-C). To investigate the role of hsa-miR-30b-5p in CXCL1-induced ICAM-1-mediated cell migration, we used a hsa-miR-30b-5p mimic. Transfection with the hsa-miR-30b-5p mimic increased the expression of hsa-miR-30b-5p and suppressed the mRNA level of ICAM-1 (Figure 7D-E). The hsa-miR-30b-5p mimic also countered the CXCL1-induced cell migration (Figure 7F). Therefore, hsa-miR-30b-5p plays a critical role in CXCL1-induced HCC cell motility by regulating ICAM-1 expression.

## Discussion

CXCL1 is a chemotactic cytokine known for its role in directing the migration of immune cells 24. It primarily affects neutrophils, CD14+ monocytes, and basophils 25. Furthermore, CXCL1 plays a critical role in extending the lifespan of neutrophils by inhibiting their apoptosis, thereby increasing their accumulation at inflammation sites 20. CXCL1 has been implicated in the progression of malignant carcinoma, affecting critical aspects such as proliferation, migration, angiogenesis, and therapy resistance 26, 27. CXCL1 contributes to angiogenesis by inducing the chemotaxis of endothelial cells 28. Furthermore, studies have shown that tumor necrosis factor (TNF) and vascular endothelial growth factor (VEGF) can stimulate the expression of CXCL1 in human lung carcinoma epithelial cells 29-31. Tumor-derived CXCL1 has been observed to promote the growth of lung cancer by attracting neutrophils from the peripheral blood into tumor tissues 32. Consistent with these findings, the circulating level of CXCL1 was found to be higher in patients with metastasis compared to patients with breast and lung cancer 32, 33. However, there is limited research on the role of CXCL1 in the metastasis of hepatocellular carcinoma. Therefore, this study mainly explores the role of CXCL1 in promoting hepatocellular carcinoma metastasis. In the present study, we investigated the correlation between the expression of CXCL1 in HCC tissue samples and the tumor stage. Furthermore, the correlation extended to patient outcomes, as higher CXCL1 expression was associated with shorter overall survival rates. HCC cell lines with greater migratory capacities exhibited higher levels of CXCL1. Our findings further confirmed that increased migratory properties are supported by elevated CXCL1 expression. Furthermore, the study demonstrates that CXCL1 actively stimulates cell migration in HCC cell lines. These findings further emphasize the significance of CXCL1 in HCC and suggest its potential as a therapeutic target.

ICAM-1 is a transmembrane glycoprotein in the Ig-like superfamily and plays critical roles in cell-cell adhesion and interaction with the extracellular matrix (ECM) 34. ICAM-1 is present in liver sinusoidal endothelial cells (LSECs), hepatocytes, Kupffer cells (KCs), and hepatic stellate cells (HSCs) 35. The gene exhibits constitutive expression in the liver and undergoes further upregulation in response to inflammatory stimuli such as TNF-α, IL-1β, or IFN-γ 36, 37. In inflammatory conditions, the liver recruits immune cell populations, such as neutrophils, lymphocytes, and monocytes. This recruitment is facilitated by the interactions between endothelial ICAM-1 and its counterpart receptor, lymphocyte function-associated antigen (LFA)-1, on lymphocytes 38, 39. The expression of ICAM-1 in various liver cells, including both parenchymal and non-parenchymal cells, enhances its potential to drive disease progression. The soluble form of ICAM-1 (sICAM-1) exacerbates the pro-metastatic phenotype, pro-inflammatory signaling, and pro-tumoral signaling 40. Elevated serum levels of sICAM-1 have been identified as a valuable marker for metastatic stage, disease recurrence, and prognosis in various types of cancer, including non-Hodgkin's lymphoma, hepatocellular carcinoma (HCC), lung cancer, and others 41, 42. In lung cancer and tongue squamous cell carcinoma cell lines, elevated expression of ICAM-1 has been linked to increased invasiveness and proliferation 37, 42. Our research revealed that CXCL1 increases ICAM-1 levels, and the utilization of ICAM-1 siRNA diminished ICAM-1 expression and reduced the CXCL1-stimulated migration in hepatocellular carcinoma cells. Additionally, reducing CXCL1 expression through CXCL1 shRNA resulted in a simultaneous decrease in ICAM-1 levels. These outcomes emphasize the critical function of ICAM-1 in the metastatic progression of hepatocellular carcinoma facilitated by CXCL1.

ICAM-1 is regulated by several microRNAs, promoting tumor cell invasion and metastasis 12, 43, 44. The dysregulated expression of miRNAs has been closely associated with processes such as tumor cell invasion and metastasis in hepatocellular carcinoma 45, 46. For instance, suppression of miR-15-5p enhances cell growth and migration by elevating levels of ICAM-1 in endothelial cells 12. However, identifying the precise microRNA responsible for regulating ICAM-1 in the migration of hepatocellular carcinoma cells remains a challenge. In our study, we used open-access prediction software to identify miRNAs that have the potential to target ICAM-1. Subsequently, we identified miR-30b-5p as a potential miRNA that can interact with ICAM-1. Transfecting cells with the miR-30b-5p mimic increased the expression of miR-30b-5p. Furthermore, the miR-30b-5p mimic significantly suppressed CXCL1-promoted ICAM-1 production and cell migration in hepatocellular carcinoma cells. These results suggest that CXCL1 promotes migration in hepatocellular carcinoma by upregulating ICAM-1 expression by inhibiting miR-30b-5p synthesis.

**Figure 8:**
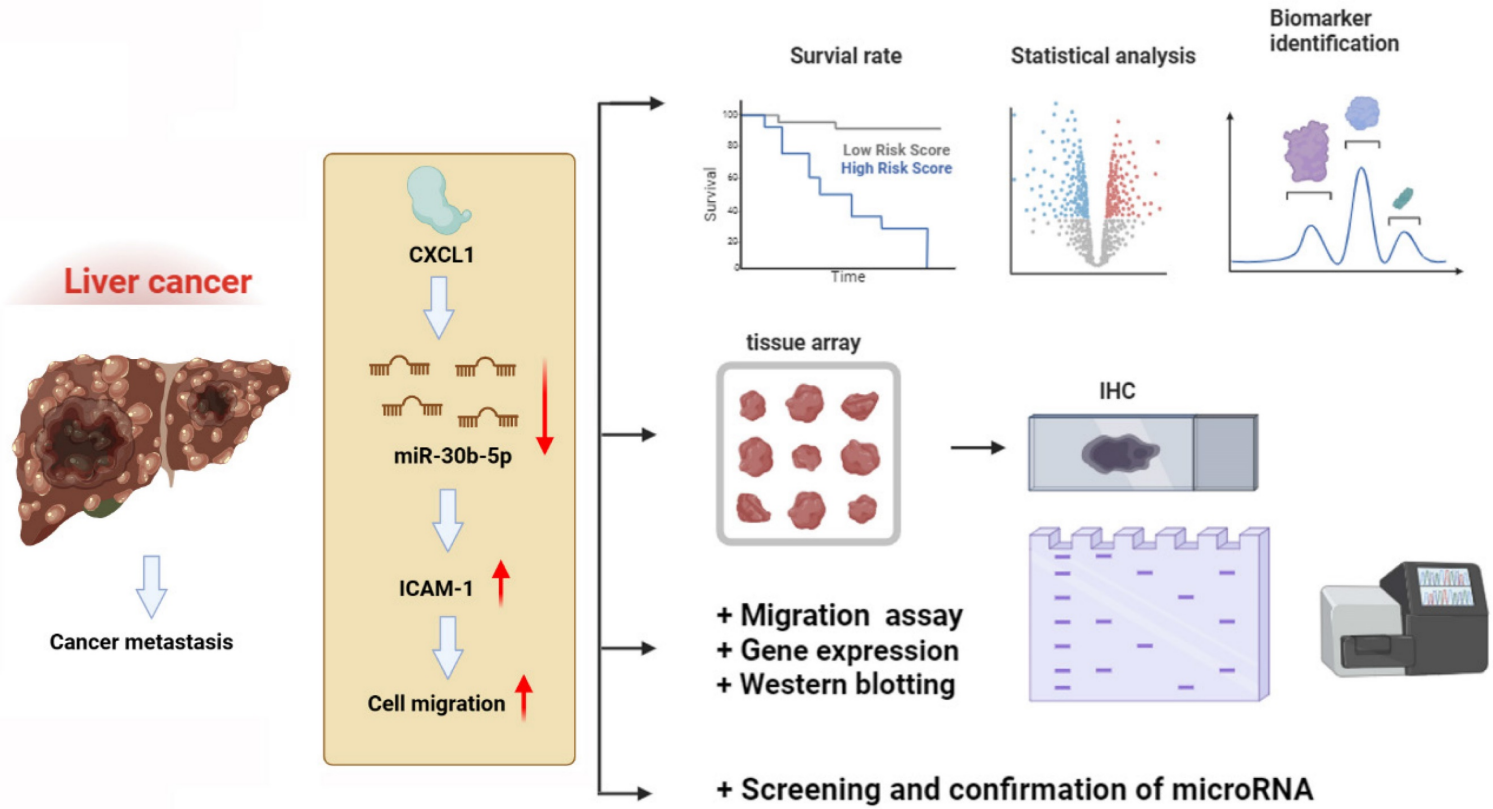
** Schematic diagram illustrating the mechanisms of CXCL1 function in hepatocellular carcinoma metastasis.** The schematic sketch summarizes the mechanisms underlying the CXCL1-induced increase in ICAM-1 production in human hepatocellular carcinoma by inhibiting miR-30b-5p generation, subsequently enhancing metastasis.

In conclusion, our findings have revealed that CXCL1 downregulates the expression of miR-30b-5p, leading to increased expression of ICAM-1, thereby promoting the motility of hepatocellular carcinoma cells (Figure 8). This discovery introduces new targeted treatment options for hepatocellular carcinoma, which could lead to the development of more effective therapeutic strategies and ultimately improve patient prognosis and survival rates.
